# Dopamine Axon Targeting in the Nucleus Accumbens in Adolescence Requires Netrin-1

**DOI:** 10.3389/fcell.2020.00487

**Published:** 2020-06-25

**Authors:** Santiago Cuesta, Dominique Nouel, Lauren M. Reynolds, Alice Morgunova, Angélica Torres-Berrío, Amanda White, Giovanni Hernandez, Helen M. Cooper, Cecilia Flores

**Affiliations:** ^1^Department of Psychiatry, Douglas Mental Health University Institute, McGill University, Montreal, QC, Canada; ^2^Integrated Program in Neuroscience, McGill University, Montreal, QC, Canada; ^3^Queensland Brain Institute, The University of Queensland, Brisbane, QLD, Australia

**Keywords:** adolescence, cortical development, guidance cues, nucleus accumbens, dopamine innervation

## Abstract

The fine arrangement of neuronal connectivity during development involves the coordinated action of guidance cues and their receptors. In adolescence, the dopamine circuitry is still developing, with mesolimbic dopamine axons undergoing target-recognition events in the nucleus accumbens (NAcc), while mesocortical projections continue to grow toward the prefrontal cortex (PFC) until adulthood. This segregation of mesolimbic versus mesocortical dopamine pathways is mediated by the guidance cue receptor DCC, which signals dopamine axons intended to innervate the NAcc to recognize this region as their final target. Whether DCC-dependent mesolimbic dopamine axon targeting in adolescence requires the action of its ligand, Netrin-1, is unknown. Here we combined shRNA strategies, quantitative analysis of pre- and post-synaptic markers of neuronal connectivity, and pharmacological manipulations to address this question. Similar to DCC levels in the ventral tegmental area, Netrin-1 expression in the NAcc is dynamic across postnatal life, transitioning from high to low expression across adolescence. Silencing Netrin-1 in the NAcc in adolescence results in an increase in the expanse of the dopamine input to the PFC in adulthood, with a corresponding increase in the number of presynaptic dopamine sites. This manipulation also results in altered dendritic spine density and morphology of medium spiny neurons in the NAcc in adulthood and in reduced sensitivity to the behavioral activating effects of the stimulant drug of abuse, amphetamine. These cellular and behavioral effects mirror those induced by *Dcc* haploinsufficiency within dopamine neurons in adolescence. Dopamine targeting in adolescence requires the complementary interaction between DCC receptors in mesolimbic dopamine axons and Netrin-1 in the NAcc. Factors regulating either DCC or Netrin-1 in adolescence can disrupt mesocorticolimbic dopamine development, rendering vulnerability or protection to phenotypes associated with psychiatric disorders.

## Introduction

**FIGURE 1:**
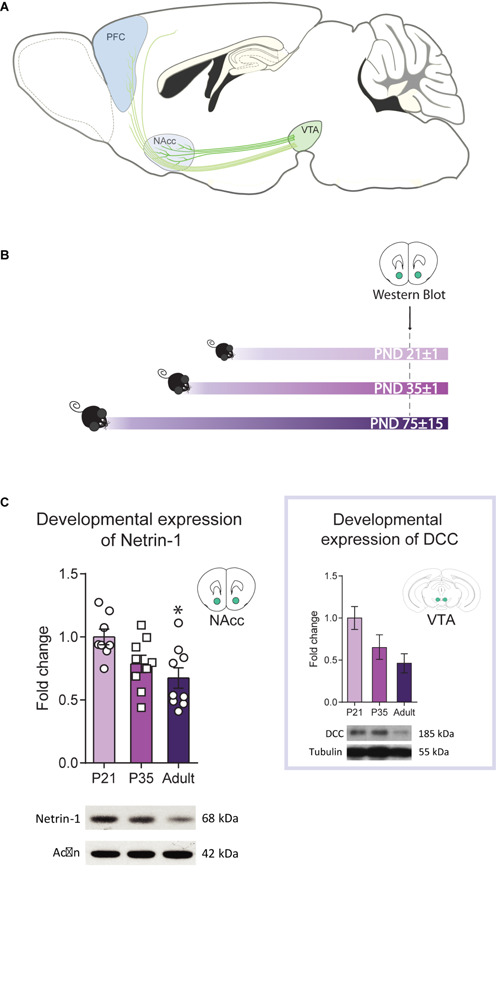
Netrin-1 levels in the nucleus accumbens (NAcc) vary across postnatal life. **(A)** Sagittal section of an adolescent mouse brain showing the mesolimbic system, composed of dopamine projection neurons that innervate the NAcc and the mesocortical circuitry, that innervate the prefrontal cortex (PFC) (image adapted from Torres-Berrio et al., 2018). Mesolimbic dopamine axons are already present in the NAcc while mesocortical dopamine axons continue to grow to the PFC across the adolescent period. The different shades of green represent DCC expression levels in dopaminergic axons and the shades of blue the expression levels of Netrin-1 in target regions. **(B)** Timeline and experimental procedures. **(C)** Levels of Netrin-1 in the NAcc of mice at three different postnatal ages. Expression decreases significantly from early adolescence to adulthood (*significantly different from adulthood, *p <* 0.05; *n* = 8–9/group). *Inset:* Data reproduced from Manitt et al. (2010) showing levels of DCC protein in the ventral tegmental area (VTA) at the at same three postnatal ages (one-way ANOVA: *F*_(2,14)_ = 3.50, *p* = 0.06). All data are shown as mean ± SEM.

Adolescence is a critical developmental period characterized by dramatic neurological and behavioral changes, with neurocircuitry in the prefrontal cortex (PFC), a brain region essential for cognitive and reward functions, being established during this time (Gogtay et al., 2004; Sowell et al., 2004; Petanjek et al., 2011). The developmental trajectory of the PFC in adolescence is demarcated by the gradual increase in dopamine innervation (Rosenberg and Lewis, 1995; Weickert et al., 2007; Rothmond et al., 2012; Gulley and Juraska, 2013; Hoops and Flores, 2017), is highly responsive to genetic and environmental factors, and determines vulnerability or resilience to psychiatric disease (Lee et al., 2014; Fuhrmann et al., 2015). The density of the mesocortical dopamine input continues to increase across adolescence due to the ongoing growth of dopamine axons toward the PFC (Hoops et al., 2018; Reynolds et al., 2018). This contrasts with the anatomically and functionally distinct mesolimbic dopamine pathway, which achieves adult-like density levels soon after postnatal development (Antonopoulos et al., 2002; Brummelte and Teuchert-Noodt, 2006). Both pathways extend from the ventral tegmental area (VTA) along the medial forebrain bundle, but segregate at the level of the striatum in a process mediated by the guidance cue receptor DCC (Figure 1A). In mesolimbic dopamine axons, DCC receptors promote target recognition events in the nucleus accumbens (NAcc) in adolescence (Reynolds et al., 2018). Mesocortical dopamine axons, however, lack or only rarely express DCC receptors and instead of recognizing the NAcc as their final target, they continue to grow to the PFC across adolescence (Manitt et al., 2011; Reynolds et al., 2018).

The DCC receptors, like other guidance cue receptors, interpret secreted soluble or cell-bound molecules in the extracellular environment that act as a signal for growing axons. The primary ligand for DCC is the guidance cue Netrin-1, which is expressed in forebrain targets of dopamine neurons, including the NAcc and dorsal striatum (Shatzmiller et al., 2008; Manitt et al., 2011; Li et al., 2014). DCC receptors may require Netrin-1 to induce dopamine targeting in adolescence because the expression pattern of these proteins in dopamine axons and forebrain post-synaptic targets is complementary (Manitt et al., 2011). In the PFC, Netrin-1 expression is substantial and localized mainly to the cortical layers that receive the densest dopamine innervation (Manitt et al., 2011), but PFC dopamine axons lack or rarely express DCC. In contrast, in the NAcc, where Netrin-1 expression is widespread but weak, DCC receptors are highly and exclusively expressed by dopamine axons (Manitt et al., 2011). A coordinated action of DCC and Netrin-1 in the development of the mesocorticolimbic dopamine system in adolescence is also suggested by findings from studies with *Netrin-1* haploinsufficiency mice. Adult mice with *Netrin-1* haploinsufficiency show increased medial PFC dopamine concentrations in comparison to wild-type mice and are protected against amphetamine-induced increase in locomotor activity similarly to adult mice with *Dcc* haploinsufficiency (Flores et al., 2005; Grant et al., 2007; Manitt et al., 2011, 2013; Pokinko et al., 2015). This idea has not been tested directly and cannot be assumed because DCC receptors also interact with ligands other than Netrin-1, including Draxin (Ahmed et al., 2011; Meli et al., 2015; Shinmyo et al., 2015; Liu et al., 2018).

Netrin-1 has long been thought to diffuse far from its source to form a gradient along which axons grow. Still, recent evidence shows that Netrin-1 binds avidly to cell surfaces and to the extracellular matrix, functioning as an adhesive cue promoting haptotaxis and fasciculation (Manitt and Kennedy, 2002; Varadarajan et al., 2017; Moreno-Bravo et al., 2019; Wu et al., 2019). Once axons reach their intended targets, Netrin-1 also plays a critical role in synapse formation (Boyer and Gupton, 2018) and in synaptic plasticity by potentiating excitatory synaptic transmission via the insertion of GluA1 AMPA receptors (Glasgow et al., 2018). All these processes require DCC-mediated Netrin-1 signaling and maybe also occurring throughout adolescence.

Here we assessed whether Netrin-1 in the NAcc is required for the adolescent maturation of the mesocorticolimbic dopamine circuitry. We first characterized the expression pattern of Netrin-1 in the NAcc across postnatal life and compared it to the dynamic expression of DCC receptors in the VTA. We then evaluated whether reducing Netrin-1 levels in the NAcc in adolescence affects the extent and the organization of dopamine connectivity in the PFC, neuronal connectivity in the NAcc itself, and behavioral responses to stimulant drugs in adulthood.

## Materials and Methods

### Animals

All experiments and procedures were performed according to the guidelines of the Canadian Council of Animal Care and the McGill University/Douglas Mental Health University Institute Animal Care Committee. C57BL/6 wild-type male mice were obtained from Charles River Canada and maintained in the colony room of the Douglas Mental Health University Institute Neurophenotyping Center on a 12-h light–dark cycle (light on at 0800 h) with food and water available ad libitum. All the experiments were conducted during the light part of the cycle.

### Drugs

d-Amphetamine sulfate (Sigma-Aldrich, Dorset, United Kingdom, Cat#A5880) was dissolved in 0.9% saline and administered i.p. at a volume of 0.1 ml/10 g and at a dose of 2.5 mg/kg of amphetamine.

### Western Blot Analysis

Mice were rapidly decapitated, and their brains were flash-frozen in 2-methylbutane (Thermo Fisher Scientific, Hampton, NH, United States) chilled with dry ice. Bilateral punches of the NAcc were excised from 1-mm-thick coronal slices starting from sections corresponding to Plate 14 of the mouse brain atlas (Franklin and Paxinos, 2007) and processed for western blot as before (Manitt et al., 2010; Cuesta et al., 2018). Briefly, protein samples (15 μg) were separated on a 10% SDS-PAGE and transferred to a nitrocellulose membrane which was incubated overnight at 4°C with antibodies against Netrin-1 (1:7500, Cat#554223, BD Pharmingen, Mississauga, ON, Canada) and β-actin (1:15,000, Sigma-Aldrich, Oakville, ON, Canada). All the samples of the experiment were run and developed in parallel. To calculate the fold change, the optical density (OD) obtained for each band of Netrin-1 was normalized using the corresponding actin OD. To normalize the data to P21, the average of the Netrin-1 OD/actin OD ratio obtained for each animal was calculated and used as a reference to normalize all the ratios obtained in the experiment.

### *Netrin-1* shRNA Expressing Lentivirus

Pre-designed and validated siRNA sequences (Ambion) were used to create shRNA by the addition of a standard hairpin loop (TTCAAGAGA) between the sense and antisense sequences. Three independent pre-designed and validated siRNA sequences against mouse *Netrin-1* were used (*Netrin-1* shRNA sequence 1: CGCCUAUCACCAAACAGAA; *Netrin-1* shRNA sequence 2: GGAGCUCUAUAAGCUAUCA; and *Netrin-1* shRNA sequence 3: UCAUCUCCGUGUACAAGCA). Three scrambled controls were created by rearranging the sequence order so that there was less than a 64% interaction rate. Active or control shRNA sequences were cloned into a pLentiLox 3.7 vector (Addgene, Plasmid #11795). Importantly, the constructs express GFP under the CMV promoter and the shRNA sequence under the U6 promoter, which allows verification of infection and visualization of the injection site for the *in vivo* experiments. The efficiency of knockdown for shRNA constructs was tested in cultured HEK cells by co-expressing a pBKNetrin-1-Flag plasmid containing the mouse *Netrin-1* cDNA cloned into a pBK-CMV vector (Stratagene) with a C-terminal Flag tag (gift of Dr. Andreas Püschel to HM Cooper), Flag expression was then evaluated by western blot using an anti-Flag antibody (1:5000; Cat#F1804; Sigma-Aldrich, Saint Louis, MO, United States). Lentiviruses expressing shRNAs and scrambled controls were prepared by the SPARC Biocentre lentiviral core facility (SickKids Hospital, Toronto, ON, Canada).

### Stereotaxic Surgery

Post-natal day (PND) 21±1 mice were anesthetized with isoflurane (5% for induction and 2% for maintenance) and placed in a stereotaxic apparatus. Simultaneous bilateral microinfusions of the lentivirus expressing an shRNA against Netrin-1 or a scrambled sequence, each with GFP, into the NAcc were performed stereotaxically using Hamilton syringes. *NAcc coordinates*: +2.6 mm (anterior/posterior), +1.5 mm (lateral), and −3.75 mm (dorsal/ventral) relative to Bregma, at a 30°. A total of 0.5 μl of purified virus was delivered on each side over an 8-min period followed by a pause of 6 min as previously (Reynolds et al., 2018). At the end of each corresponding experiment, all the infected mice were euthanized for immunohistochemistry to verify the site of microinjection via evaluation of GFP expression.

### Neuroanatomical Analysis

#### Perfusion

Adult mice received an intraperitoneal overdose of ketamine 50 mg/kg, xylazine 5 mg/kg and acepromazine 1 mg/kg and were perfused intracardially with 50 ml of 0.9% saline followed by 75 ml of chilled fixative solution (4% paraformaldehyde in phosphate-buffered saline). Brains were dissected and placed in the fixative solution overnight at 4°C and were then transferred to phosphate-buffered saline and stored for a maximum of 2 days. Brains were sectioned using a vibratome (35-μm-thick coronal slices for medial PFC analysis and 100 μm thick coronal slices for spine morphology analysis).

#### Immunofluorescence

Every second coronal section was processed (1:2 series) as previously reported (Manitt et al., 2010, 2011, 2013). A rabbit polyclonal anti-tyrosine hydroxylase (TH) antibody (1:1000 dilution, catalog #AB152; Millipore Bioscience Research Reagents) and an Alexa Fluor 594-conjugated secondary antibody raised in goat (1:500 dilution, 1 h incubation, Invitrogen) were used.

#### Stereology

The TH antibody selected labels dopamine axons in the PFC with high specificity, and rarely labels norepinephrine axons (Miner et al., 2003; Manitt et al., 2011, 2013). As previously, and because of the lateralization of the dopamine system, we obtained counts only from the right hemisphere. To evaluate changes in dopamine connectivity in animals with reduced levels of Netrin-1 in the NAcc during adolescence, we performed stereological quantification of the span of TH-positive fibers in the cingulate 1, prelimbic, and infralimbic subregions of the pregenual medial PFC (Figure 3B). The specific role of mesocortical dopamine function appears to vary across these medial PFC subregions (Kalivas, 2008; Peters et al., 2009; Van De Werd et al., 2010) and in the past we have found developmental changes in dopamine connectivity to be more pronounced across the ventral or dorsal axis (e.g., Reynolds et al., 2015). The total volume of TH-positive fiber innervation (in cubic micrometers) was assessed using the Cavalieri method using Stereoinvestigator^®^ (MicroBrightField) (Manitt et al., 2011, 2013). To determine the density of TH-positive varicosities, we used the optical fractionator probe of Stereoinvestigator^®^ (MicroBrightField) (Manitt et al., 2011, 2013). The coefficient of error was below 0.1 for all regions of interest in all sampled brains. Counts were performed blind.

#### Medial PFC Analysis

The medial PFC subregions were delineated according to plates spanning 14–18 of the mouse brain atlas (Franklin and Paxinos, 2007). A 5× magnification was used to trace the contours of the dense TH-positive innervation of the subregions using a Leica DM400B microscope (Figure 3B). An unbiased counting frame (25 × 25 μm) was superimposed on each contour and counts were made at regular predetermined intervals (*x* = 175 μm, *y* = 175 μm) from a random start point. Counting of varicosities was performed at ×100 magnification on 5 of the 12 sections contained within the rostrocaudal borders of our region of interest (Plates 14–18; 1:4 series). A guard zone of 5 μm was used and the optical dissector height was set to 10 μm.

### Analysis of Spine Density and Morphology

Dendrites from GFP-positive neurons contained in the NAcc were systematically selected for imaging fluorescence, using a confocal microscope (Olympus FV 1200) at 60× immersion objective at 4× zoom and 1024 × 1024 pixels resolution. The Z stacks acquisition were performed at 0.3 μm increments. For each mouse, approximately 5–8 dendritic segments were quantified using NeuronStudio software^1^. Only dendritic segments from both hemispheres with at least 10 μm of length and that were clearly distinguishable from other segments were included in the analysis. NeuronStudio determines dendrite length semi-automatically and classifies individual spines into thin, mushroom, or stubby according to (i) spine aspect ratios, (ii) head-to-neck diameter ratios, and (iii) head diameters. Thin spines have a neck ratio value (head to neck diameter ratio) less than 1.1 and a length to spine head diameter greater than 2.0. Mushroom spines have a neck ratio value above 1.1 and a spine head diameter equal or greater than 0.3 μm. Stubby spines were discernable by the lack of neck. Each dendritic segment was analyzed separately according to the number of spines and the diameter of the head spines. The density and head diameter were calculated per 10 μm of dendritic length. The analysis was performed by an experimenter fully blinded across groups.

### Acute Amphetamine Behavioral Response

Locomotor activity was quantified as before (Reynolds et al., 2015) using an infrared activity monitoring apparatus modified for use with mice (AccuScan Instruments, Columbus, OH, United States). Locomotion was measured as distance traveled (cm). Stereotypy counts were measured as the number of breaks of the same photocell beam or set of beams repeatedly as defined by the AccuScan system. D-amphetamine sulfate salt was dissolved in 0.9% saline (2.5 mg/kg) and injected intraperitoneally (i.p.) to adult mice microinfused with the shRNA for Netrin-1 or the scrambled sequence into the NAcc during adolescence.

### Statistical Analysis

All statistical analyses were performed using Prism 6 for Windows (GraphPad Software, La Jolla, CA, United States). All values are represented as means ± S.E.M. A significance threshold of α = 0.05 was used in all the experiments. Statistical differences between two groups were analyzed with Student’s *t*-tests. All data are normally distributed, and the variance is similar between groups. Statistical differences between more than two groups were analyzed with one-way or two-way ANOVAs, followed by Bonferroni multiple comparison post hoc tests. The sample size in all the experiments varied from 4 to 8 animals per group.

## Results

### Dynamic Postnatal Developmental Expression of Netrin-1 Expression in the NAcc

We have shown previously that *Dcc* mRNA and protein expression in the VTA decreases from adolescence to adulthood (Manitt et al., 2010; Cuesta et al., 2018). If Netrin-1 in the NAcc contributes to DCC-dependent dopamine axon targeting in adolescence, its expression may follow a similar expression pattern to DCC. We measured Netrin-1 expression in the NAcc of mice at PND21±1, PND35±1, and PND75±15 (Figure 1B) and found that the levels are high in early adolescence, but diminish significantly by adulthood (Figure 1C; one-way ANOVA: *F*_(2,23__)_ = 5.20, *p* = 0.014; the expression at PND21 is significantly higher than PND75; post hoc Bonferroni, *p* < 0.05). The similarity between Netrin-1 (Figure 1B) and DCC protein expression patterns (Figure 1C *inset*, one-way ANOVA: *F*_(2,14)_ = 3.50, *p* = 0.06, adapted from Manitt et al., 2010) during postnatal life suggests that coordinated regulatory mechanisms titrate the expression of this receptor-ligand pair.

### Silencing Netrin-1 Expression *in vivo*

**FIGURE 2:**
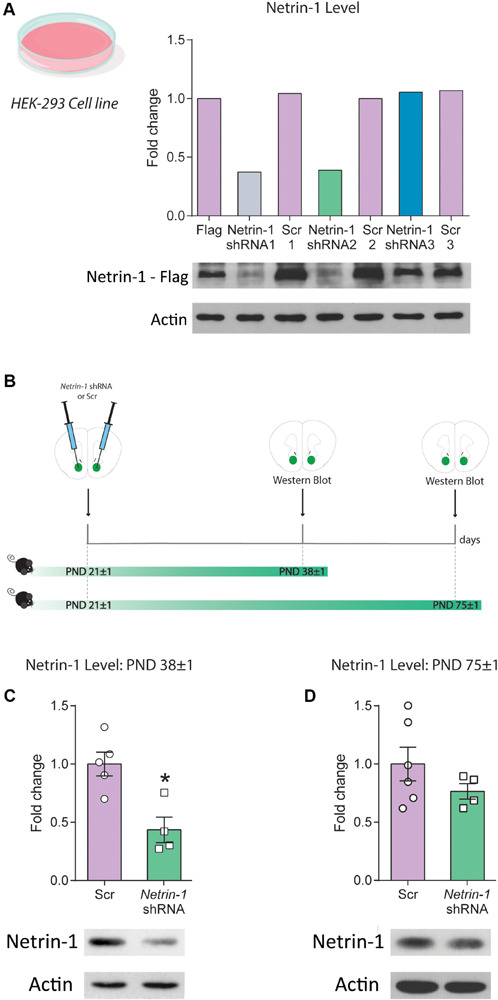
Downregulation of Netrin-1 protein expression in adolescence *in vivo*. **(A)** HEK293 cells transfected to express a mouse Netrin-1 protein with a C-terminal Flag tag were co-transfected with three different shRNA constructs (*Netrin-1* shRNA 1, 2, and 3), their corresponding Scrambled (Scr) sequences (Scr 1, 2 and 3) or mock transfected. Only *Netrin-1* shRNA constructs 1 and 2 downregulated Netrin-1 protein expression by more than 50% in comparison to cells transfected with the corresponding scrambled sequence or the mock transfected Netrin-1-Flag expressing cells. This finding was replicated in two separate experiments. **(B)** Timeline and experimental procedures for *in vivo* regulation of Netrin-1 protein expression. **(C)** Significant downregulation of Netrin-1 protein expression in the nucleus accumbens (NAcc) 2 weeks after microinfusing a lentiviral construct containing the *Netrin-1* shRNA sequence (*significantly different from Scrambled; *p* = 0.007; *n* = 4–5/group). **(D)** *Netrin-1* shRNA-mediated downregulation in adolescence does not alter Netrin-1 protein expression in adulthood (*n* = 4–6/group). All data are shown as mean ± SEM.

We tested the efficiency of three different viral constructs expressing shRNA sequences against *Netrin-1* (*Netrin-1* shRNA 1–3) and their corresponding scrambled sequences (Scrambled 1–3) using cultured HEK cells that co-expressed a mouse Netrin-1 protein with a C-terminal Flag tag (Figure 2A). Infection with the *Netrin-1* shRNA constructs differentially downregulated Netrin-1 expression in comparison to the Netrin-1-Flag construct and the scrambled sequences. However, of the three constructs we designed, only constructs 1 and 2 downregulated Netrin-1 expression by more than 50%. These findings were replicated in a second separate experiment (data not shown).

We packaged the shRNA construct 2 (henceforth referred to as “*Netrin-1* shRNA”) into a lentivirus and injected it bilaterally into the NAcc of P21 mice. Control mice received bilateral microinfusions of the corresponding scrambled sequence (Figure 2B). Netrin-1 protein expression was reduced (∼60%) in the NAcc 2 weeks following *Netrin-1* shRNA microinfusions, in comparison to control groups (Figure 2C, *t*_(7)_ = 3.76, *p* = 0.007). This effect is transient because Netrin-1 protein expression in the NAcc of adult mice that received *Netrin-1* shRNA or scrambled construct microinfusions in early adolescence do not differ (Figure 2D, *t*_(8)_ = 1.25, *p* = 0.25).

### Netrin-1 Downregulation in the Nacc During Adolescence Disrupts Pfc Da Connectivity

**FIGURE 3:**
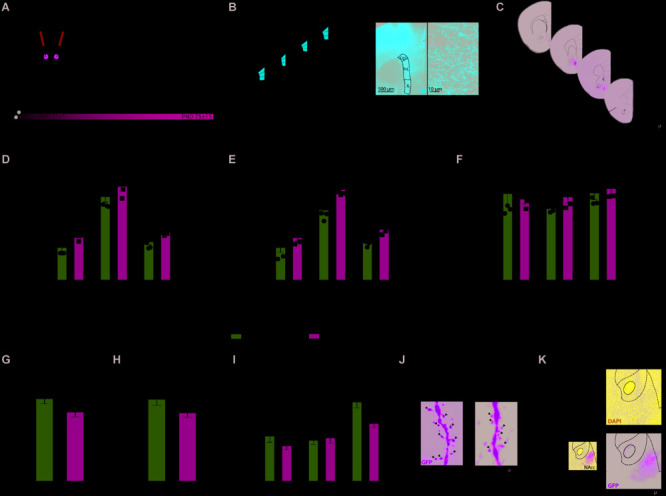
Netrin-1 downregulation in the nucleus accumbens (NAcc) during adolescence disrupts dopamine connectivity in the adult prefrontal cortex (PFC) and alters NAcc dendritic spine structure in adulthood. **(A)** Timeline of treatment and experimental procedures. **(B)** Schematic representation of the regions of interest in the medial PFC outlined according to the Mouse Brain Atlas (Franklin and Paxinos, 2007). The cingulate (Cg1), prelimbic (PrL), and infralimbic (IL) subregions of the medial PFC were analyzed. Left panels: (i) micrograph of a coronal section of the pregenual medial PFC at a low magnification (×5) showing an overlay of the contours traced to delineate subregions of interest; (ii) micrograph of a coronal section of the pregenual mPFC a high magnification (×100) showing the tyrosine hydroxylase (TH)-positive varicosities (adapted from Reynolds et al., 2015). **(C)** Representative serial micrographs of an adult mouse showing lentiviral infection in the NAcc. Adult mice microinfused with a lentivirus containing the *Netrin-1* shRNA in the NAcc in adolescence have **(D)** increased dopamine input volume **(E)** and total number of TH-positive varicosities in comparison to their shRNA scrambled microinfused counterparts (*significantly different from Scrambled microinfused mice, *p <* 0.05). **(F)** There are no differences in TH-positive varicosities between groups. *n* = 4–5/group. Netrin-1 downregulation in the NAcc in adolescence leads to local structural changes in medium spiny neurons (MSN) in adulthood. Specifically, in comparison to Scrambled, *Netrin-1* shRNA induces **(G)** a reduction in dendritic spine density, **(H)** a trend toward decreased spine head diameter (*p* = 0.07), and **(I)** a decrease in mushroom-type spines (*significantly different from Scrambled microinfused mice, *p <* 0.05) *n* = 3-4/group. **(J)** Representative images of dendritic segments of MSN from a Scrambled- or shRNA- injected mice. White arrows indicate mushroom spines. **(K)** High magnification picture of an injection site stained with nuclear marker DAPI reveals not significant cells loss in the infected area. All data are shown as mean ± SEM.

We used unbiased stereology to assess whether the downregulation of Netrin-1 in the NAcc during adolescence affects PFC dopamine connectivity in adulthood (Figures 3A–C). We found a significant increase in the span (i.e., volume) of the dopamine input across the three subregions of the PFC, infralimbic, prelimbic and cingulate in adult mice microinfused with the *Netrin-1* shRNA construct in adolescence, in comparison to scrambled controls (Figure 3D: two-way ANOVA, significant main effect of virus microinfusion, *F*_(1,7)_ = 9.09, *p* = 0.0195; no significant virus microinfusion × medial PFC region interaction, *F*_(2,14)_ = 0.117, *p* = 0.89; significant main effect of medial PFC region, *F*_(2,14)_ = 139.1, *p* < 0.0001). This effect is accompanied by a significant increase in the total number of dopamine varicosities in *Netrin-1* shRNA mice versus controls (Figure 3E two-way ANOVA, significant main effect of virus microinfusion, *F*_(1,7)_ = 14.23, *p* = 0.007; no significant virus microinfusion × medial PFC region interaction, *F*_(2,14)_ = 0.173, *p* = 0.21; significant main effect of medial PFC region, *F*_(2,14)_ = 118.5, *p* < 0.0001, *post hoc* Bonferroni, significant difference in the prelimbic subregion between *Netrin-1* shRNA- and Scrambled-infused mice, *p* = 0.002). There are no differences between *Netrin-1* shRNA and scrambled groups in the density of PFC TH-positive varicosities (Figure 3F: two-way ANOVA, no significant main effect of virus microinfusion, *F*_(1,7)_ = 0.63, *p* = 0.45; no significant virus microinfusion × medial PFC region interaction, *F*_(2,14)_ = 0.58, *p* = 0.57; no significant main effect of medial PFC region, *F*_(2,14)_ = 1.10, *p* = 0.36). These anatomical changes are similar to those observed in adult mice with downregulation of DCC receptors in dopamine axons in adolescence, suggesting that Netrin-1 signaling through DCC receptors is required for mesolimbic dopamine axon targeting in the NAcc (Manitt et al., 2011, 2013; Reynolds et al., 2018). There was no visible tissue damage present in the site of infection (Figure 3K), which is consistent with previous reports (Naldini et al., 1996; Blömer et al., 1997; Davidson and Breakefield, 2003; Ahmed et al., 2004).

### Silencing Netrin-1 in the NAcc in Adolescence Remodels Spine Morphology in Adulthood

Netrin-1 promotes synapse formation in the PFC (Goldman et al., 2013) and potentiates excitatory synaptic transmission in the hippocampus via the insertion of GluA1 AMPA receptors in adult mice (Glasgow et al., 2018). We analyzed the dendritic segments of this neurons in adult mice bilaterally microinfused with either *Netrin-1* shRNA or Scrambled virus into the NAcc at P21 (Figure 3A). Total dendritic spine density is significantly reduced in the *Netrin-1* shRNA group when compared with Scrambled controls (Figure 3G: *t*_(41)_ = 2.04, *p* = 0.048) and there is a significant reduction in the density of mushroom spines in the *Netrin-1* shRNA group compared to Scrambled-infused mice (Figures 3I,J: two-way ANOVA, significant main effect of virus microinfusion, *F*_(1,41)_ = 4.26, *p* = 0.045; significant virus microinfusion × spine type interaction, *F*_(2,82)_ = 4.38, *p* = 0.016; significant main effect of spine type, *F*_(2,82)_ = 29.86, *p* < 0.0001, *post hoc* Bonferroni, significant difference in mushroom spines between *Netrin-1* shRNA- and Scrambled-infused mice, *p* = 0.0038). While not significant, we also observed a trend toward a reduction in the total head diameter (Figure 3H: *t*_(41)_ = 1.82, *p* = 0.076).

### Reduced Sensitivity to the Locomotor Effects of Amphetamine in Adulthood as a Consequence of Silencing Netrin-1 in Adolescence

**FIGURE 4:**
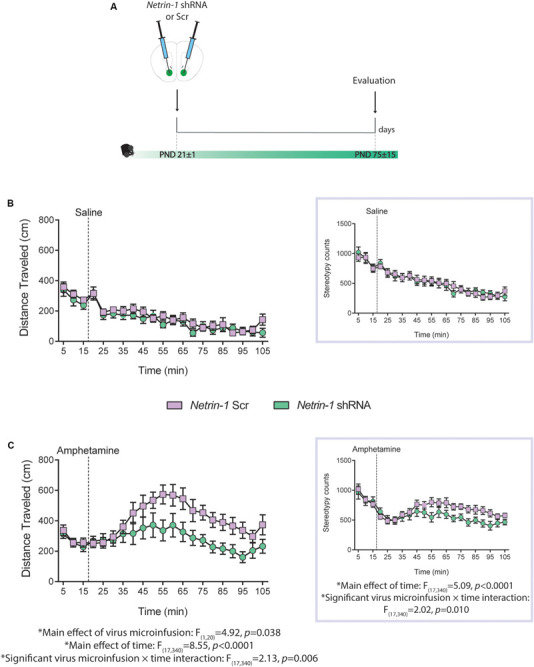
Netrin-1 downregulation in the nucleus accumbens (NAcc) in adolescence leads to reduced sensitivity to amphetamine in adulthood. **(A)** Timeline of drug treatment and experimental procedures. Distance traveled and total stereotypy *(inset)* after **(B)** a saline i.p. injection or **(C)** an amphetamine i.p. injection (2.5 mg/kg). *n* = 10–12/group. All data are shown as mean ± SEM.

We have shown that reduced *Dcc* expression in dopamine neurons in adolescence leads to blunted locomotor response to amphetamine in adulthood (Manitt et al., 2013), and that this effect results from increased dopamine input to the PFC (Pokinko et al., 2015). Here we investigated whether silencing Netrin-1 in the NAcc in adolescence would lead to a similar phenotype. P21 mice were microinfused in the NAcc with the *Netrin-1* shRNA or Scrambled lentivirus. In adulthood, all mice were administered an i.p. injection of saline or amphetamine (2.5 mg/kg; Figure 4A). There are no group differences in locomotor activity or in stereotypy counts following i.p. saline administration (Figure 4B, distance traveled: no significant main effect of virus microinfusion: *F*_(1,20)_ = 0.80, *p* = 0.38, no significant virus microinfusion × time interaction: *F*_(17,340)_ = 0.79, *p* = 0.70; significant main effect of time: *F*_(17,340)_ = 13.8, *p* < 0.0001; stereotype counts *(inset)*: no significant main effect of virus microinfusion: *F*_(1,20)_ = 3.78, *p* = 0.066, significant virus microinfusion × time interaction: *F*_(17,340)_ = 2.02, *p* = 0.010, significant main effect of time: *F*_(17,340)_ = 5.09, *p* < 0.0001). However, mice that had received *Netrin-1* shRNA infusions in adolescence show reduced amphetamine-induced locomotion and stereotypy counts in adulthood, when compared to Scrambled controls (Figure 4C; distance traveled: significant main effect of virus microinfusion: *F*_(1,20)_ = 4.92, *p* = 0.038, significant virus microinfusion × time interaction: *F*_(17,340)_ = 2.13, *p* = 0.006; significant main effect of time: *F*_(17,340)_ = 8.55, *p* < 0.0001; stereotype counts *(inset):* no significant main effect of virus microinfusion: *F*_(1,20)_ = 3.78, *p* = 0.066, significant virus microinfusion × time interaction: *F*_(17,340)_ = 2.02, *p* = 0.010, significant main effect of time: *F*_(17,340)_ = 5.09, *p* < 0.0001).

## Discussion

The fine arrangement of neuronal connectivity during development involves the coordinated action of guidance cues and their receptors. DCC receptors within VTA dopamine neurons are crucial for the targeting of their mesolimbic projections to the NAcc in adolescence and for delimiting the extent of their input to the PFC (Manitt et al., 2013; Reynolds et al., 2018). Here we assessed whether dopamine axon targeting in adolescence also requires Netrin-1 expression in the NAcc. We find that similar to the expression of DCC in the VTA, Netrin-1 levels in the NAcc are dynamic across postnatal life, with levels transitioning from high to low in adolescence. Netrin-1 downregulation, specifically in the NAcc during adolescence, results in an increased expanse of PFC dopamine innervation, in altered spine morphology of NAcc medium spiny neurons (MSNs), and in reduced sensitivity to the behavioral effects of stimulant drugs of abuse in adulthood. These developmental changes and adult behavioral outcomes mimic the phenotypes induced by *Dcc* haploinsufficiency, strongly suggesting that the interaction between DCC receptors in dopamine axons and Netrin-1 in the NAcc is required for the appropriate establishment of mesocorticolimbic dopamine circuitry in adolescence.

We have previously observed a high-to-low complementary expression of Netrin-1 in the NAcc and of DCC receptors in mesolimbic dopamine axons during adolescence and adulthood that suggested a direct interaction of DCC and Netrin-1 signaling (Manitt et al., 2011). The similarity in the postnatal expression patterns of Netrin-1 in the NAcc and DCC in the VTA support this idea and raise the possibility that the regulation of ligand and receptor levels may involve a common mechanism and may be required for mesolimbic DA axons to recognize the NAcc as their final target (Reynolds et al., 2015, 2018).

Spines of MSN in the NAcc emerge after the second postnatal week, with peak spine density occurring around the third postnatal week in rodents (Antonopoulos et al., 2002). Netrin-1 in the NAcc appears to participate in this process because silencing Netrin-1 in the NAcc at PND21, produces a decrease in spine density in adulthood. Netrin-1 has been shown to promote dendritic arbor complexity (Goldman et al., 2013) and to facilitate the microenvironment that stimulates filopodia extension and synaptogenesis (Colon-Ramos et al., 2007; Shen and Cowan, 2010; Poon et al., 2013). This process seems to involve alterations in the organization of the actin cytoskeleton (Hlushchenko et al., 2016), the clustering of pre- and post-synaptic proteins via Src kinase signaling and m-Tor-dependent protein translation (Goldman et al., 2013), and the activation of the JNK1/c-Jun signaling pathway (Zheng et al., 2018). The Rho family of small GTPases, Rac1 and Cdc42, is an integral component of the signaling pathway that regulates spine morphogenesis (Luo et al., 1996; Luo, 2000; Nakayama et al., 2000). Netrin-1/DCC interactions are known to activate both Rac1- and Cdc42-mediated actin polymerization (De Vries and Cooper, 2008; see Lanoue and Cooper, 2019 for an extensive review).

The overall reduction in spine density after Netrin-1 downregulation appears to be driven by a reduction in the density of mushroom spines, which are required for synapse maturation and stabilization (Matsuzaki et al., 2001; Berry and Nedivi, 2017). Mushroom spines contain the largest excitatory synapses and contain AMPA receptors (Zito et al., 2009). The reduction of mushroom spines suggests that viral-mediated silencing of Netrin-1 disrupts the mechanisms by which AMPA receptors are recruited. The structural plasticity of dendrites decreases as circuits mature. During adolescence (P21–P60), changes ongoing in dendrite branches begin to stabilize in rodents, while a fraction of dendritic spines remain dynamic, with a net loss of spines (Koleske, 2013). In contrast to early timepoints, it is possible that Netrin-1 expression in the NAcc becomes more important for maintaining the stabilized/matured (mushroom) spines at the beginning of adolescence. In this case, Netrin-1 downregulation in the NAcc in adolescence could lead to a net loss of this type of spines without affecting the stability or number of immature thin spines.

Adult mice microinfused with the *Netrin-1* shRNA in adolescence show diminished locomotor response to a challenge injection of the stimulant drug amphetamine. The increase in PFC dopamine connectivity is likely underlying these behavioral effects, considering that mesocortical dopamine neurotransmission exerts inhibitory control over mesolimbic dopamine activity (Grace, 1991; Le Moal and Simon, 1991; Ventura et al., 2004; Pokinko et al., 2015). The increase in PFC dopamine innervation observed in mice treated with the *Netrin-1* shRNA likely prevents amphetamine-induced dopamine release into the NAcc, resulting in the observed blunted drug locomotor response. This idea is consistent with the behavioral phenotype displayed by adult mice with *Netrin-1* or with *Dcc* haploinsufficiency (Grant et al., 2007; Pokinko et al., 2015). These mice show reduced locomotor activity in response to amphetamine and this effect is restored following 6-OHDA intra-PFC injections (Pokinko et al., 2015). We cannot rule out that alterations in synaptic organization at the level of the NAcc may account for the behavioral phenotype observed in adult mice with *Netrin-1* shRNA infusions in adolescence. MSN receive multiple excitatory synaptic inputs via their numerous spines. A reduction in spine density and altered shape may render MSNs less excitable, affecting NAcc-mediated behaviors, including drug-induced locomotor activity. Indeed, dopamine and excitatory inputs converge onto MSN and mediate behavioral and incentive motivational effects of amphetamine and other psychostimulants (Swanson and Kalivas, 2000; Sesack et al., 2003; Li et al., 2011).

## Conclusion

In summary, our findings indicate that the complementary interaction between DCC receptors expressed by dopamine axons and Netrin-1 expressed by NAcc neurons and located in the surrounding extracellular matrix is required for the developmental organization of the mesocorticolimbic dopamine circuitry. Environmental risk or protective factors for psychopathology may impact the development of this system by regulating DCC and/or Netrin-1 expression in adolescence. Adolescent exposure to recreational-like doses of amphetamine [i.e., doses that reach peak plasma concentrations within the range of those seen in recreational use (Cuesta et al., 2019)] downregulates both DCC in dopamine neurons and Netrin-1 in the NAcc, disrupting the development of dopamine circuitry and of cognitive control (Yetnikoff et al., 2010; Reynolds et al., 2015, 2018; Cuesta et al., 2018, 2019; Reynolds and Flores, 2019). In contrast, therapeutic-like doses of amphetamine [i.e., doses that reach peak plasma concentrations within the range of those observed in therapeutic settings (Cuesta et al., 2019)] upregulate DCC protein expression without altering Netrin-1, leading to protective-like phenotypes. Interventions for adolescents are currently lacking (Das et al., 2016). Targeting DCC or Netrin-1 may serve as a novel preventive/treatment strategy for youth.

## Data Availability Statement

The raw data supporting the conclusions of this article will be made available by the authors, without undue reservation, to any qualified researcher.

## Ethics Statement

All the animals experiments and procedures were performed according to the guidelines of the Canadian Council of Animal Care and the McGill University/Douglas Mental Health University Institute Animal Care Committee.

## Author Contributions

SC and CF conceived and designed the experiments, analyzed the results, and wrote the manuscript. SC performed all the molecular evaluations, the developmental characterization, drug experiments, and performed stereotaxic surgeries. DN performed all the neuroanatomical analysis. LR and AW developed the different plasmid constructs. LR provided technical training for anatomical analysis and contributed to the editing of the manuscript. AM performed stereotaxic surgeries. AT-B contributed in tissue collection. GH contributed to the writing and editing of the manuscript. HC generated the lentiviral constructs and provided technical training necessary to perform the research. All authors contributed to the article and approved the submitted version.

## Conflict of Interest

The authors declare that the research was conducted in the absence of any commercial or financial relationships that could be construed as a potential conflict of interest.

## Footnotes

1 http://research.mssm.edu/cnic/tools-ns.html
